# Skin‐Inspired Thermoreceptors‐Based Electronic Skin for Biomimicking Thermal Pain Reflexes

**DOI:** 10.1002/advs.202201525

**Published:** 2022-07-25

**Authors:** João Neto, Radu Chirila, Abhishek Singh Dahiya, Adamos Christou, Dhayalan Shakthivel, Ravinder Dahiya

**Affiliations:** ^1^ Bendable Electronics and Sensing Technologies (BEST) Group University of Glasgow Glasgow G12 8QQ UK

**Keywords:** artificial thermoreceptors, dielectrophoresis, electronic skins, nanowires, printed electronics, temperature sensors, vanadium pentoxide

## Abstract

Electronic systems possessing skin‐like morphology and functionalities (electronic skins [e‐skins]) have attracted considerable attention in recent years to provide sensory or haptic feedback in growing areas such as robotics, prosthetics, and interactive systems. However, the main focus thus far has been on the distributed pressure or force sensors. Herein a thermoreceptive e‐skin with biological systems like functionality is presented. The soft, distributed, and highly sensitive miniaturized (≈700 µm^2^) artificial thermoreceptors (ATRs) in the e‐skin are developed using an innovative fabrication route that involves dielectrophoretic assembly of oriented vanadium pentoxide nanowires at defined locations and high‐resolution electrohydrodynamic printing. Inspired from the skin morphology, the ATRs are embedded in a thermally insulating soft nanosilica/epoxy polymeric layer and yet they exhibit excellent thermal sensitivity (−1.1 ± 0.3% °C^−1^), fast response (≈1s), exceptional stability (negligible hysteresis for >5 h operation), and mechanical durability (up to 10 000 bending and twisting loading cycles). Finally, the developed e‐skin is integrated on the fingertip of a robotic hand and a biological system like reflex is demonstrated in response to temperature stimuli via localized learning at the hardware level.

## Graphical abstract

This work presents anelectronic skin (e‐skin) having distributed, miniaturized temperature sensors or thermoreceptors and a peripheral nervoussystem‐like localized learning scheme, allowing robots to mimic the thermal pain like reflexes. The vanadium pentoxide nanowires (NWs)‐based thermoreceptors are developed with dielectrophoresis approach for precise assembly and high‐resolution printing of NWs. The e‐skin exhibits excellent thermal sensitivity, fast response, and flexibility.

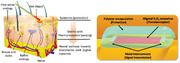

## 1 Introduction

**Figure 1:**
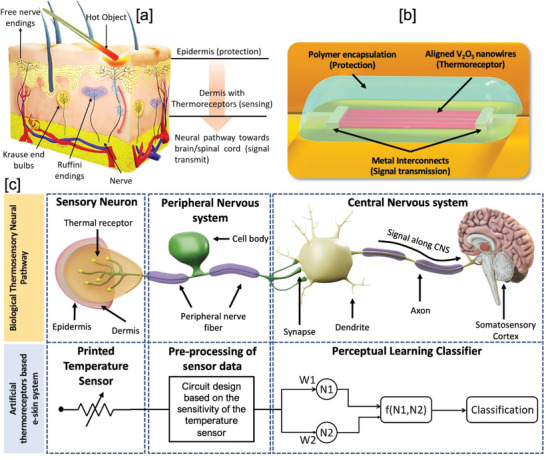
The biological and artificial thermal sensory neural pathway. a) Human‐skin model showing the buried free nerve endings, thermoreceptors, and nociceptors under the epidermis as protection layer. b) The developed artificial thermoreceptors (ATRs) where the thermosensitive V_2_O_5_ NWs are buried under soft polymer layer mimicking the architecture of biological thermoreceptors. c) The biological thermal sensory neural pathway and the conceptual framework for creating its artificial equivalent. The connection for the artificial neural pathway is for illustration purpose only.

Biological skin detects wide range of mechanical and thermal stimuli^[^ ^1^ ^]^ to trigger protective reflexes^[^ ^2^ ^]^ and similar touch sensory feedback‐based functions are being sought for robotics,^[^ ^3^ ^]^ prosthetics,^[^ ^4^ ^]^ and interactive systems.^[^ ^5^ ^]^ Electronic skins (e‐skins)^[^ ^6^ ^]^ have attracted significant attention in recent years, with main focus on distributed pressure or force sensors only. However, the ability to perceive other stimuli such as temperature is equally crucial. It facilitates to maintain a constant body temperature and reaction to painful hot or cold objects. The pain feeling is established by an activation of sensory neurons when exposed to pernicious thermal (extreme heat [>52 °C]^[^ ^7^ ^]^ or cold [<5 °C]^[^ ^8^ ^]^) stimuli. The spatially distributed thermoreceptors, nociceptors, and nerve endings in the skin sense and transmit this critical information to the brain for perception and suitable action (**Figure** **1**). The functions of these receptors inspire the development of robots and artificial limbs with pain‐related reflexes and advance the field that has so far focused on mimicking the mechanoreceptors for pressure or force feedback.^[^ ^3^, ^5^, ^6^, ^9^ ^]^ Interestingly the pain related reflexes have not caught the attention of roboticists, despite the fact that the studies elucidating the mechanism of noxious stimuli (mechanical and thermal) related reflexes were reported more than two decades (for which the 2021 Nobel prize in medicine was also awarded).^[^ ^7^, ^8^, ^10^ ^]^ The artificial skin mimicking the morphology and processing capabilities of biological thermosensory pathway has not been explored so far and only few reports^[^ ^6b^ ^]^ available in this area are because of: 1) the precise neural pathway for thermal sensing mechanism has remained elusive, 2) the lack of heat‐sensitive and robust soft materials, and 3) the technological challenges related to processing of soft materials on flexible substrates over large areas.

The recent advances in printed electronics have opened interesting opportunities for cost‐effective and resource‐efficient fabrication^[^ ^11^ ^]^ and various printed heat‐sensitive soft materials investigated to‐date are summarized in Table S1, Supporting Information. Among these, the electrically conductive nanocomposites with conductive fillers (inorganic/carbonaceous) in viscoelastic polymer matrix (organic) have been explored extensively.^[^ ^12^ ^]^ These sensors demonstrate excellent flexibility and high sensitivities, but they also exhibit high thermal hysteresis, considerable inaccuracy during cyclic heating and cooling, and operate in narrow sensing range. As a result, the reported solutions have restricted applicability for biological systems like reflexes over a wide temperature stimuli.^[^ ^13^ ^]^ For example, the perception of cold feelings has not been demonstrated so far. Further, unlike the miniaturized and distributed thermoreceptors in biological skin, the demonstrated nanocomposite‐based devices are fairly big in size. Recreating the architecture and functionality of biological thermoreceptors requires miniaturized distributed sensors that possess excellent sensitivity, wide sensing range, stable and accurate response (robust), and flexibility. Considering these requirements, the 1D inorganic nanowires (NWs) can be excellent building block for the development of temperature sensitive e‐skin.^[^ ^14^ ^]^ Despite such a potential, the NW‐based skin electronics has not matured as it is challenging to integrate them at well‐defined locations with high reproducibility and uniformity. Additive printing via nozzle‐based methods such as inkjet, and extrusion (direct‐ink writing) have gained attention for the development of e‐skin.^[^ ^15^ ^]^ These printing methods allow patterning of conductive materials such as liquid metals,^[^ ^15b,c^ ^]^ and colloidal metal nanoparticles^[^ ^15a^ ^]^ in a resource‐efficient manner. Whilst impressive progress has been made in compliant electrical systems with interconnects based on these materials offering superior electrical and mechanical properties, it is challenging to define high resolution electrodes from them using printing technologies over 1D materials due to issues such as: 1) ink spreading, 2) difficulties in getting precise control over the geometry of liquid metal patterns, and 3) poor printing resolutions.

To mimic the thermal sensing neural pathway, the e‐skin must also efficiently handle a large amount of sensing data with low‐latency so that faster reflexes can be attained and potential damages to humans or prosthetic/robotic devices can be prevented. The data processing for perceptual experience is currently performed using software program that adapt and learn independently.^[^ ^16^ ^]^ However, this is an inefficient approach as encoding, processing, and transmission of a large amount of data affects the latency. The active perception with on‐chip peripheral nervous system like localized learning at the hardware level could mitigate such issues. Driven by these motivations, herein, we report an artificial thermoreceptor (ATR)‐based e‐skin system to mimic a biological system like sensing and reflex action (excitatory and inhibitory) in response to wide temperature stimuli (5–360 °C).

## 2 Results and Discussions

### 2.1 Fabrication of Temperature Sensors and Material Characterization

**Figure 2:**
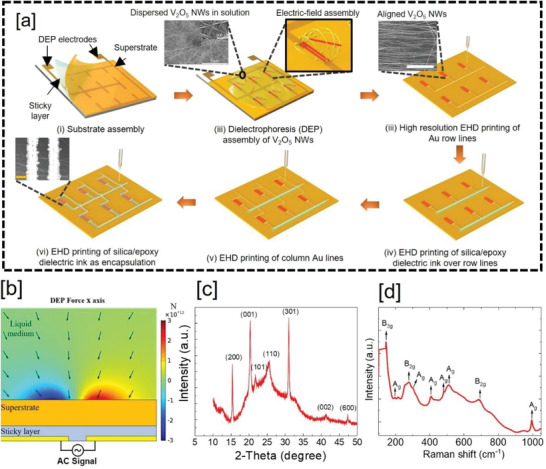
a) Schematics/SEM images illustrating the fabrication of artificial thermoreceptors using DEP aligned V_2_O_5_ NWs: (i) Superstate (substrate on which NWs are aligned) attached over DEP electrodes using thin double side tape. (ii) DEP process for precise and oriented assembly of NWs. Inset (left) shows SEM image of the entangled NWs (as purchased) and inset (right) shows electric field lines to trap NWs. (iii) EHD printing to define Au metal electrodes and interconnects (row lines). Inset shows the magnified SEM image of the aligned NWs. (iv) EHD printing of NS/epoxy dielectric over row lines of the sensor matrix. (v) EHD printing to define Au electrodes and interconnects (column lines). (vi) Final encapsulation step using NS/epoxy defined over sensing channel and column lines. Inset shows magnified SEM image of single unit of the fabricated sensor matrix. b) Simulated DEP force according to the *x*‐axis cross sectioning the DEP electrodes. The DEP force is shown in red and blue color and electric field gradient plot is shown by arrows. c) The XRD 2‐theta scan, and d) Raman spectra for the V_2_O_5_ NWs.

Figure 1c schematically illustrates the thermosensory neural pathway in human skin, which consists of thermoreceptors that receive heat/cold signals from the environment and transmit to the neural network. These thermal receptors show dynamic as well as static excitatory or inhibitory responses to represent the magnitude and rate of change of cold and hot stimuli. Thus, presented thermosensory system in the fabricated e‐skin prototype is capable of behaving as ATRs after perceptual learning. The prototype maps the functionality of thermal sensory neural pathway in the human body for the data encoding, transmission, and processing. The temperature sensors are fabricated with vanadium pentoxide (V_2_O_5_) NWs as a heat‐sensitive layer, and electrohydrodynamic (EHD) printing to deposit high‐resolution metal/dielectric lines defining the channel length, interconnect structures, and encapsulation. The fabrication process for the sensors is illustrated schematically in **Figure** **2**a (see Experimental Section and Supporting Information for details). The randomly dispersed V_2_O_5_ NWs in deionised (DI) water are deterministically placed with a position and orientation‐controlled assembly over large areas using dielectrophoresis (DEP) technique (Figure 2a[ii]). The fabrication of high‐performance NW‐based skin electronics demands highly aligned structures to reduce the electron scattering at the NW–NW junction and to facilitate the efficient charge transport.^[^ ^17^ ^]^ In the present case, this feature is needed to achieve fast and uniform response from the printed temperature sensors. Achieving such conformations through DEP requires a proper analysis of different process variables influencing both the NW density and alignment quality. Toward this, COMSOL simulations are performed to optimize the DEP assembly process (Figure 2b) where forces in the order of pN are necessary to attract, align, and place the NWs at the defined locations. The details of the COMSOL simulation are described in Experimental Section and Figure S1a–c and Note S1, Supporting Information. During DEP, the amplitude of an applied voltage plays a critical role to control the NW density at each assembly site. For instance, at 40 V_PP_, the generated field gradient is not strong enough to initiate the NW assembly. At 100 V_PP_ the DEP force is stronger enough for NW trapping and alignment (see Figure S2a, Supporting Information). At 180 V_PP_, we observed further increase in NW density. At higher voltages, the DEP force is strong enough to attract the NWs further from the assembly site. The higher DEP forces also decrease the NW–surface separation to a distance at which van der Waals forces dominate (≈10 nm), and the NWs closely conform to the surface.^[^ ^18^ ^]^ The optimized DEP parameters, that is, 180 V_PP_ and 500 kHz, lead to a NW density of ≈9 ± 1.5 NW/µm at each assembly site (see Figure S2b,c, Supporting Information). It is evident from the Figure S2b,c, Supporting Information, that the optimized DEP parameters resulted in aligned NW structures. Next, EHD printing was carried out to complete the temperature sensor fabrication process.

Before printing the metal electrodes over the NWs (Figure 2a[iii,v]) and dielectric materials (Figure 2a[iv,vi]), the EHD technique was optimized over polyimide (PI) substrate to achieve uniform and superfine printed lines with resolution of 5 µm (see Experimental Section and Figure S3 and Note S2, Supporting Information). The structural quality of the sensing material, that is, V_2_O_5_ NWs was examined using X‐ray diffraction (XRD) (Figure 2c) and Raman spectroscopy (Figure 2d). From the XRD data, diffraction peaks can be indexed to an orthorhombic V_2_O_5_ phase.^[^ ^19^ ^]^ No characteristic peaks related to any impurities or related to any other V_2_O_5_ phases such as monoclinic are detected in this pattern. The Raman peaks located at 144, 195, 281, 305, 407, 480, 510, 694, and 994 cm^−1^ can be assigned to the Raman signature of V_2_O_5_.^[^ ^19^, ^20^ ^]^ The Raman peaks at 144 and 995 cm^−1^ hint the layered structure of V_2_O_5_ where distorted trigonal bipyramidal atoms are stacked up and share edges to form (V_2_O_4_)_n_ zigzag double chains along the [001] direction and are cross‐linked along the [100] direction through the shared corners.^[^ ^20^ ^]^ The dominant charge transport in such a layered structured V_2_O_5_ is through hopping mechanism which is required to achieve high temperature sensitivity.^[^ ^21^ ^]^ In addition, the Raman peaks at about 305, 407, 510, and 694 cm^−1^ are assigned to the bending vibration of V—O_C_ (A_g_ mode), the bending vibration of V—O_B_—V bond (A_g_ mode), the stretching vibration of V—O_B_—V bond (A_g_ mode), and the stretching vibration of V—O_C_ bond (B_2g_ mode), respectively.^[^ ^20^ ^]^

Both XRD and Raman characterization tools have been used to study the oxygen defects in V_2_O_5_ and thus, the presence of V^4+^/V^5+^.^[^ ^19a^ ^]^ These impurity centers assist in hopping of electrons and define the conductivity in V_2_O_5_ (discussed later in details) and sensor response. Whilst the role of oxygen defects sites for gas sensing is well‐understood, their role in temperature sensing is unclear. The position of (001) XRD diffraction peak at 2*θ* of 21.3° of the V_2_O_5_ NWs could hint the presence of oxygen vacancies in the material. It was experimentally observed that with an increase in oxygen vacancies there is a shift in (001) peak to a larger angle (21.43°).^[^ ^19a^ ^]^ The presence of (001) diffraction peak at 21.3° in the present case indicates low level of oxygen vacancies. Raman spectroscopy is generally used for the microscale analysis of the material and can provide information on the location of oxygen deficiency in V_2_O_5_ nanostructures. It is known that there are four types of oxygen sites (O(I), O(II), O(III), and O(IV)) bonded with one V atom in a single layer of the layered V_2_O_5_ orthorhombic structure.^[^ ^19a^ ^]^ The Raman peak at 480 cm^−1^ arises from bending vibration of V—O(II) bridge, peak at 526 cm^−1^ is associated with stretching vibration of V—O(IV), the vibrations of V=O(I) is related with the peak intensity at 993 cm^−1^, and V–O(III) with the peak at 696 cm^−1^. The strong and distinct peaks appearing at 696 and 993 cm^−1^ in the Raman spectrum suggest that the oxygen vacancy sites are mainly at the bridging O(I) and O(II) sites of the V_2_O_5_ NWs in the present case.^[^ ^19a^ ^]^

### 2.2 Electrical and Electromechanical Characterizations of Temperature Sensors

**Figure 3:**
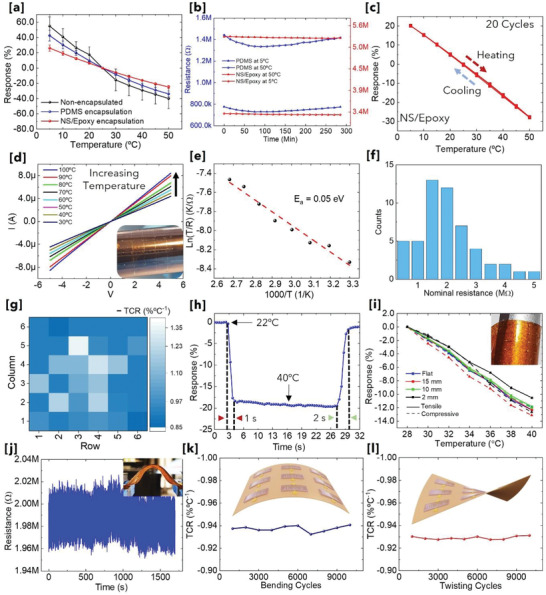
Electrical, sensing, and electromechanical performance of ATRs: a) Comparison of sensor response (%) against temperature ranging from 5 to 50 °C for non‐encapsulated and encapsulated devices. Two different encapsulation materials are tested, namely NS/epoxy (printed) and PDMS (spin‐coated). For each device prototype, that is, non‐encapsulated, PDMS‐encapsulated, and NS/epoxy‐encapsulated, the data was obtained from five devices to have average value with error bars. b) Long‐term (≈5 h) heating–cooling cycles to monitor resistance variation at 5 and 50 °C for NS/epoxy and PDMS‐encapsulated devices. The data shows small drift for PDMS‐encapsulated devices. The devices with NS/epoxy encapsulation showed more stable response. c) Forward and reverse sensing data from 5 to 50 °C (multiple cycles) with calculated response (%) showing negligible hysteresis with NS/epoxy encapsulation. The hysteresis was negligible to be visible in the presented graph. Electrical and sensing performance for sensor encapsulated with NS/epoxy: d) Temperature‐dependent electrical (*I*–*V*) transport properties of V_2_O_5_ NWs; e) Plot of ln(*T*/*R*) versus 1000/*T* (data extracted from the *I*–*V* results shown in [b]); f) Statistical diagram of nominal resistance variation for randomly selected 52 devices; g) The spatial distribution graph of the sensitivity from 36 devices to investigate the uniformity of the sensor response. h) NS/epoxy encapsulated sensor response when transferred from 22 to 40 °C, showing fast response (1 s) and recovery (2 s). Electromechanical sensor response: i) Response (%) of the sensor against temperature ranging from 28 to 40 °C under various bending conditions; j) Resistance variation for >1000 continuous bending cycles; k) Variation in TCR with different numbers of bending cycles (bending radius = 10 mm). The inset shows a schematic of the sensor matrix under bending loading; l) Variation in TCR with different numbers of twisting cycles (±30°) showing excellent durability. The inset shows a schematic of the sensor matrix under twisting.

The performance of the as‐fabricated temperature sensing device (non‐encapsulated) was first evaluated for a typical human‐sensing range of 5–50 °C. The change in resistance with time was monitored for every 5 °C step change in temperature (see Figure S4a, Supporting Information). Two continuous cycles were performed to obtain the data needed to evaluate the device stability (where one cycle represents temperature change from 25 to 5 °C then to 50 °C and back to 25 °C). The data exhibits poor device stability (large hysteresis) and non‐linear (*R* ^2^ ≈ 0.9) response (see Figure S4b, Supporting Information). We hypothesize that the observed non‐linearity for non‐encapsulated devices could be due to the influence of humidity and condensation of water vapors at lower temperatures (see Figure S4c,d, Supporting Information). This is because of the observed large resistance change at lower temperatures (for similar change in temperature steps of 5 °C)—at which condensed water molecules were observed (see Figure S4e, Supporting Information). To prove this, we carried out further experiments with another non‐encapsulated device under different relative humidity (RH). While the nominal resistance of this device is slightly higher, the sensor's sensitivity is in the similar range. A custom‐made experimental set‐up was used to control the RH while measuring the change in resistance of the device (see Figure S4c,d, Supporting Information). It is evident from Figure S4d, Supporting Information, that under low RH (20%), the non‐encapsulated device showed stable and more linear response as compared to the one shown in Figure S4a, Supporting Information. When we increased the RH to 50%, there is a rapid decrease in the sensor's resistance (most likely due to additional adsorption of oxygen and water molecules on the NW surface and depletion of free charges).^[^ ^22^ ^]^ However, the sensor was still operational, showing distinct resistance step change with temperature. With further increase in RH (nearly 100%), a rapid increase in the device conductivity was observed and the sensor showed no response to step change in temperatures. A plausible explanation to the observed device response under different RH to change in temperatures is as follows: In our view there is a trade‐off between two physical processes affecting the electrical properties of the V_2_O_5_ NWs. The first one is depletion of free electrons from the NW surface due to adsorption of water and oxygen molecules (leading to increase in device resistance)^[^ ^22^ ^]^ and the second relates to freely moving protons at higher‐level physiosorbed water layers according to Grotthuss mechanism, which leads to decrease in device resistance.^[^ ^23^ ^]^ When exposed to low humidity ambient, the low concentration of water molecules gets adsorbed onto the NW surface. In this case, the depletion process dominates as the protons are too far apart (due to low concentration) which leads to increase in the device resistance. At this stage, conductivity is defined by electron transport. At higher humidity levels protonic conductivity dominates over the electronic one, thereby enhancing the conductivity of the V_2_O_5_‐based sensor.^[^ ^23a^ ^]^ As a result of this change in the charge transport mechanism under very high RH conditions, the sensor's resistance varied randomly with temperature. Further studies are on‐going to confirm this hypothesis. Nevertheless, inspired from the biological skin, the fabricated devices are buried under a soft polymeric layer to improve the device robustness and softness. Two different polymers namely, polydimethylsiloxane (PDMS) and nanosilica/epoxy (NS/epoxy) were tested as encapsulation layers. This also prevents metals from eroding/corroding, strengthens the mechanical robustness, and reduces the effect of moisture/humidity. The changes in electrical resistance were monitored for the non‐encapsulated devices as well as those encapsulated with PDMS (see Figure S5a,b, Supporting Information) and NS/epoxy (see Figure S5c,d, Supporting Information). Both encapsulated devices showed more linear response (*R* ^2^ for PDMS‐encapsulated devices is 0.98 and *R* ^2^ for NS/epoxy encapsulated is 0.99) with lesser hysteresis as compared to non‐encapsulated devices (*R* ^2^ ≈ 0.9) measured under similar conditions. The rate of change of the sensor resistance (response [%]) was extracted from the electrical data and plotted for all three device variants, that is, without encapsulation, with spin‐coated PDMS, and EHD‐printed NS/epoxy dielectric (**Figure** **3**a). It should be noted that five devices were measured for each of three device variants. The sensor's sensitivity can be calculated using sensor response in percent ((Δ*R*/*R*_0_) × 100) change with temperature where Δ*R* = *R* − *R*_0_ (*R* is the resistance at temperature *T* and *R*_0_ is the nominal resistance at room temperature). The temperature coefficient of resistance (TCR) was further extracted from the slope of the fitted linear line for all the variants shown in Figure 3a (see Note S3, Supporting Information, for TCR calculation details).^[^ ^24^ ^]^ The TCR decreased progressively from non‐encapsulated (≈−2.1% °C^−1^) to PDMS coated (≈−1.7% °C^−1^) and to NS/epoxy coated (≈−1.1% °C^−1^) temperature sensors. The decrease in TCR/sensitivity with encapsulation could be because of lesser impact of ambient conditions. For example, it is well known that the water adsorption can significantly lower the sensitivity of metal oxide sensors.^[^ ^22^ ^]^ Likewise, prolonged exposure to humid environments could lead to the gradual formation of stable chemisorbed OH^−^ on the metal oxide surface,^[^ ^22^, ^25^ ^]^ causing a progressive deterioration of the sensitivity of the sensors. In our case, the observed decrease in thermistor sensitivity with polymer encapsulation could also be due to moisture barrier properties of polymeric encapsulants. It is known that the adsorbed water molecules dissociate into H^+^ or H_3_O^+^ ions on the metal oxides surface.^[^ ^26^ ^]^ The conduction between these dissociated ions occurs through hopping between adjacent hydroxyl groups.^[^ ^22^ ^]^ This extra path provided for hopping of charges could result in higher TCR for non‐encapsulated devices (at lower temperature larger response was observed for non‐encapsulated devices due to water condensation). But the adsorption process changes dynamically with the temperature, and this results in non‐linearity in response as well as large hysteresis for non‐encapsulated devices. The PDMS‐encapsulated devices showed slightly higher average TCR values, but more hysteresis and non‐linearity as compared to NS/epoxy coated ones. This might be because of the trapped air/water vapors in PDMS. In situ plasma treatment was performed before EHD printing of NS/epoxy over V_2_O_5_ NWs to avoid air/water molecules trapping at the interface and thus, improve the stability. Next, long term cyclic heating–cooling tests (5–50 °C) were performed to further confirm the effect of encapsulation on device stability (see Figure S6a,b, Supporting Information). The extracted data shows excellent stability of the devices with NS/epoxy polymer as an encapsulant (see Figure S6b, Supporting Information, and Figure 3b). The extracted resistance values at 5 and 50 °C showed negligible variation for up to 5 h of continuous cycles, and thus, negligible hysteresis for NS/epoxy devices (Figure 3c). The sensitivity of the NS/epoxy encapsulated devices showed decrease in sensitivity but is sufficient and robust enough to quantify a step‐change of as small as 0.3 °C (see Figure S7, Supporting Information). Accordingly, we have selected printed NS/epoxy as an encapsulation layer for all further experiments.

The temperature‐dependent *I*–*V* characteristics were performed in ambient dark conditions (Figure 3d) over a range of 30–100 °C. This was carried out to investigate the quality of printed Au and V_2_O_5_ NWs metal‐semiconductor (MS) contact, the charge transfer mechanism, and to extract the activation energy. The linear relationship with symmetric response indicates uniform MS contact along the NWs with an Ohmic behavior of Au–V_2_O_5_ NWs. Temperature‐dependent *I*–*V* measurements revealed an increase in conductivity with increasing temperature, which is consistent with thermally activated hopping transport^[^ ^20a^ ^]^ (see Note S4, Supporting Information). The Arrhenius plot for extraction of activation energy is shown in Figure 3e (*V*_D_ = 0.5 V). From the slope of the fitted linear line, the activation energy of 0.05 ± 0.01 eV was calculated. This value is a little smaller than previously reported single or multi‐NW device and significantly smaller than the numbers for film or wire network devices.^[^ ^20^, ^21^, ^27^ ^]^ Smaller activation energy can be due the presence of highly aligned NW structures, which reduces the electron scattering at the NW–NW junction, and the Ohmic MS contacts. The printed sensors based on the DEP aligned NWs with NS/epoxy encapsulated devices showing a 100% yield (for a 4 × 4 sensor matrix, Figure S8a–c, Supporting Information). Profilometer measurements showed that the thickness of printed NS/epoxy was ≈1 µm (see Figure S8d,e, Supporting Information). Finally, to confirm the efficacy of printed NS/epoxy as encapsulant layer, we tested the device under various RH conditions (similar to the experiments performed on non‐encapsulated device). The data obtained at various RH values showed devices having no major variation in hysteresis and response linearity (see Figure S9, Supporting Information).

Further, we fabricated 10 × 10 sensor matrix to evaluate the large area processability of the adopted fabrication approach. Out of the 100 devices, we randomly selected more than 50 devices to examine the device‐to‐device variation in nominal resistance (Figure 3f). Resistance distribution of the devices is relatively concentrated (1–2 MΩ). It should be noted that the devices at the edges of the sample gave slightly more variations in the nominal resistance. Next, we examined the device‐to‐device variation in the sensitivity. For this, we selected 36 devices in center of the sample and measured them under similar conditions (Figure 3g). A modest variation in device‐to‐device sensitivity (≈−1.1 ± 0.3) was observed and this could be attributed to the variation in NW density and dimensions of EHD‐printed Au lines. It is challenging to obtain similar number of NWs at each sensing site using DEP process. For better uniformity, more work is needed to decrease the NW density variations. The printed sensors were also tested beyond the skin‐sensing temperature range (see Figure S10a, Supporting Information). The temperature sensitivity for different temperature regions was extracted from the linear fitting the data points. Three different operating regimes can be observed for the measured temperature range (5–380 °C). The extracted TCR progressively decreases from 5–50 °C range (≈−1.1) to 50–140 °C (≈−0.52) and to 140–380 °C (≈−0.1). Figure 3h–l shows the experiments related to the response/recovery time and electromechanical measurements. The measured response/recovery time of the sensor showed similar values for different temperature range (see Figure S10b,c, Supporting Information). An almost instantaneous response of ≈1s was registered (Figure 3h). The fast response is attributed to the thin printed NS/epoxy encapsulant layer with a thickness of 1–2 µm as compared with PDMS which resulted in response time of ≈3 s (see Figure S10d,e, Supporting Information). The printed sensor with NS/epoxy encapsulation also showed a fast resistance recovery (≈2 s). The mechanical robustness and device stability of the fabricated temperature sensors were evaluated under different bending conditions. The sensors were subjected to tension and compression by mounting them on to a 3D printed convex and concave structures. For both bending types, the radius of bending curvature was progressively reduced from 15 to 10 to 2 mm. The temperature sensor showed small variation in sensitivity when tested under mechanical bending. This could be due to the strain‐induced modification of the V_2_O_5_ electrical properties. Irrespective of this, the sensor continues to show similar TCR value, which is attributed to the intrinsic flexibility of extremely high aspect ratio V_2_O_5_ NWs. This allows the NWs to accommodate high strain. Further, encapsulation may prevent performance deterioration as mechanical stress associated with flexible sensors could be exploited.^[^ ^28^ ^]^ We have also performed cyclic bending test to further prove the robustness of the sensor. The nominal resistance of the sensor did not vary significantly even after repeated 1000 bending cycles (maximum bending radius of 2 mm) (Figure 3j). This shows the robustness of the electrical contacts realized using EHD printing. Last, the durability and robustness of the sensors were examined by extending the number of loading cycles and type of loading. For these tests, full automated bending, and twisting endurance setup (Yuasa Ltd.) was used (see Figure S11, Supporting Information). First, the sensitivity was measured after every 1000 bending cycles up to 10 000 cycles (Figure 3k). The sensitivity of the sensor remained almost same after repeated bending strain (bending radius 10 mm). Moreover, Figure 3l shows the sensitivity change as a function of twisting cycles (±30°). As shown, no device failure was observed up to 10 000 twist cycles.

### 2.3 Thermal Sensing Neural Pathway Capable of In‐Skin Learning

**Figure 4:**
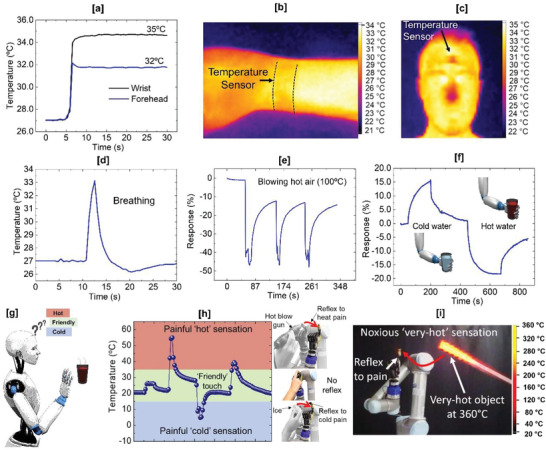
The demonstration of the artificial thermal sensing neural pathway capable of “in‐skin” learning: a) Measurement of temperatures of a human forehead and arm. b,c) The measured temperature was confirmed using commercial infrared thermometer. d) Temperature monitoring of exhaled human breath during respiration. e) Real‐time response of the sensor attached to the robotic finger when heated using hot air gun. f) Real‐time sensor response attached to the robotic to discriminate between cold and hot water in a cup. g) Schematic illustration of a robot hand approaching toward object with unknown temperature and sensing at the same time. h) Experimental demonstration of the robot's reaction toward three temperature classes: human‐friendly touch, painful hot, and cold feelings. The photos showing the acquired pain reflex after associative learning on the skin. i) Robot's hurriedly reflex to very painful sensations when a pre‐heated red‐hot rod at 360 °C approached toward its hand.

Humans exhibit great adaptability to changing thermal environment based on the previous experience (e.g., learning). In this section, functionalities such as thermosensation and thermal nociception of the biological thermal sensory system is achieved with the fabricated temperature sensors and supervised learning and their working as ATR is demonstrated (**Figure** **4**). First, the e‐skin was firmly attached to the wrist and forehead of a volunteer to measure the body temperature. An infrared thermometer was also used to confirm the accuracy of measured temperature. As shown Figure 4a–c, the distinct changes in resistance were detected from 32 °C (forehead) and 35 °C (wrist) and these were also confirmed with commercial infrared thermometer. Next, the sensor was tested to capture the temperature during respiration. The exhaled human breath during respiration is clearly recorded showing temperature ≈33 °C (Figure 4d). These data sets show that the sensor is sensitive enough to capture the real‐time changes in human body temperature and respiration. This can help predict the cognitive status of body and also provide a means for early diagnosis of diseases.^[^ ^29^ ^]^ To endow the bio‐like feeling to robots with presented e‐skin, the thermal sensing performance was evaluated on a robotic platform and sufficient data set was generated for training. Owing to the thin device substrate and flexible active materials, temperature sensor can be conformally attached to the fingertip of the commercial i‐Limb robotic/prosthetic hand. The sensor was tested under hot (using hot air gun ≈100 °C) and cold (using ice at ≈5 °C) conditions. As shown in Figure 4e, the sensor showed a sharp increase in the response (reaching ≈45%) upon blowing hot air onto the sensor. Multiple heating–cooling cycles were performed to confirm the repeatability of the sensor response. The sensor was also able to differentiate cold and hot water in a cup grasped by the robot (Figure 4f). Finally, we also show that the temperature sensing performance of presented e‐skin remained uninfluenced by the external pressure (see Figure S12, Supporting Information). The sensor responded identically when ice cubes of different weights were placed over the sensor, verifying its capability to detect the temperature regardless of the external pressure.

Most of the previously demonstrated temperature sensors for artificial skin have focused on exhibiting superior temperature sensitivity,^[^ ^29a^ ^]^ enhanced stretchability of the sensors,^[^ ^30^ ^]^ etc. However, these limited studies are insufficient as the large amount of sensing data, acquired from the artificial skin, still needs to be preprocessed, encoded, transmitted—just as the biological skin. Herein, we show that the sensitive temperature sensors can provide skin‐like perception in response to external thermal stimuli. Using the perceptual learning at the hardware‐level, we demonstrate an artificial neuromorphic thermal processing system with dynamic excitatory or inhibitory responses which represent the magnitude and rate of change of external temperature stimuli (see Note S5, Supporting Information, for details). Using the trained neural classifier, we first demonstrate the excitatory/inhibitory response for the change of the magnitude of external temperature stimuli (Figure 4g,h). For this, the conformally attached sensor on the fingertip of the commercial i‐Limb robotic hand was touched with the human finger and no response was recorded by the robotic arm (inhibitory response). This could be termed as a “human‐friendly” touch for the robots working collaboratively with human worker in an Industry 4.0 setting. On the other hand, excitatory robotic reflexes were observed for painful touch of an ice and/or hot air blown with a gun (temperature was set‐up at 100 °C) (Movie S1, Supporting Information). Next, excitatory reflexes representing the rate change of external temperature stimuli were recorded (Movie S2, Supporting Information). For this scenario, hot air gun was made to approach the sensor in a controlled manner (slower and faster). As can be seen in the Movie S2, Supporting Information, the robotic hand responded in an unhurried manner when the hot‐air gun approached slowly, whereas the robot responded quickly when the gun approach was faster. Last, excitatory reflexes were recorded when a red‐hot rod (≈360 °C) was brought closer to the robotic hand (Figure 4i). This time the robot moved away very fast, mimicking the reflexes for harsh and adverse temperature conditions (Movie S3, Supporting Information).

## 3 Conclusions

We have reported a novel e‐skin thermal sensing system allowing the robots to feel, learn, and react to external heat stimuli over wide range. The novel printed electronics route was adopted to develop the e‐skin that mimics the architecture and functionality of biological thermal sensing systems. To this end, the DEP technique was employed for deterministic, position‐, and orientation‐controlled assembly of heat‐sensitive V_2_O_5_ NWs over large areas. Furthermore, EHD printing with resolution well within few micrometers, was used for patterning printed metal electrodes, interconnects, probing pads, and dielectric (as an encapsulant). The nanosilica/epoxy (NS/epoxy) printed dielectric is selected to encapsulate the sensing channel and electrodes/interconnects in analogy with the human thermoreceptors. The resulted miniaturized, distributed, and embedded sensors showed stable, robust thermal sensing characteristics needed to mimic the biological thermoregulatory system. Finally, by integrating the e‐skin on the fingertip of a robotic hand and using localized learning at the hardware level, both dynamic excitatory and inhibitory responses, representing the magnitude and rate change of external temperature stimuli for three different temperature classes, were presented. The developed system showed inhibitory reflexes to human‐touch and excitatory reflexes to painful hot/cold, and very painful hot sensations. Compared to the previous demonstration of the flexible temperature sensors, the work presented here fully mimics the neurological principles, thus showing the capability of localized learning directly on the hardware level. Such a distributed learning through hardware holds great potential for the next‐generation robots and interactive systems that require reduced cognitive load on their central control units.

## 4 Experimental Section

### Fabrication of Temperature Sensors

The fabrication process was initiated by having a 50 µm thick PI substrate (Dupont) over the carrier substrate (glass, where the DEP electrodes were pre‐patterned), using an ultra‐thin double‐sided tape (≈5 µm thick) (Nitto) (Figure 2a[i]). The V_2_O_5_ NWs were aligned on the PI substrate. The PI was chosen as it suits the requirements for both the process and the application of sensor, where a high glass transition temperature (>350 °C) was required along with good conformability and thermal insulating property.^[^ ^31^ ^]^ In the next step, V_2_O_5_ NWs were assembled over large areas using DEP technique (details are provided in the Note S1, Supporting Information). The left inset of the Figure 2a[ii]) shows the scanning electron microscopy (SEM) image of randomly dispersed NWs in DI water while the right one shows the electric field lines to assemble the aligned NWs. The V_2_O_5_ NWs were purchased from Novarials (≈40 nm diameter, and >100 µm in length). Figure S2c, Supporting Information, shows both low‐ and high‐magnification SEM images of the as‐received aligned V_2_O_5_ NWs. The NWs had smooth surface and were 50–80 nm in diameter. Majority of NWs were several micrometers in length, resulting in an aspect ratio >2500. Next, EHD printing was used to finalize the temperature sensor fabrication. First, Au electrodes were realized to define the channel length. Then, NS/epoxy ink was printed for encapsulating the sensing channel length and electrodes/interconnects. For assessment of the sensing devices, long printed lines were used as wire bonding. Copper wires were bonded using flexible silver epoxy resin EJ2189 from Epoxy Technology Inc. The curing process was performed at 80 °C for 2 h to improve the robustness and conductivity. Then the flexible epoxy 310M from Epoxy Technology Inc. was used to package the cured silver epoxy paste. The flexible epoxy was cured at 70 °C for 1 h.

### Material Characterization

The morphology of the received V_2_O_5_ NWs was studied through SEM SU82400 from Hitachi. The nanostructure crystallinity was studied using XRD with Cu K*α*_1_ radiation on the Bruker D8 Venture kappa geometry diffractometer equipped with a Photon‐II CPAD detector. The scans were performed in the 2*θ* range from 10° to 50° at a scanning rate of 0.02° s^−1^. Room‐temperature Raman spectra of as‐received V_2_O_5_ NWs were obtained using a LabRAM HR system (Horiba Jobin Yvon). An excitation wavelength of 532 nm and a power <1 mW was used. A lens of 100× magnification was used to focus the laser beam and to collect the scattered light dispersed by a holographic grating with 2400 lines/mm.

### Electrical, Temperature Sensing, and Mechanical Bending Characterizations

Electrical characterizations of fabricated temperature sensors on the flexible PI substrate were performed in the ambient environment using Cascade Micro‐tech Auto‐guard probe station interfaced to a semiconductor parameter analyzer (B1500A, Agilent). Linkam PE120 Peltier system was used for heating and cooling the fabricated temperature sensors to perform temperature sensing measurements. The mechanical loading (bending and torsional) was applied using commercial system (Yuasa System DMLHP‐TW).

## Conflict of Interest

The authors declare no conflict of interest.

## Supporting information

Supporting Information

Supplemental Movie 1

Supplemental Movie 2

Supplemental Movie 3

## Data Availability

The data that support the findings of this study are available from the corresponding author upon reasonable request.
