# Supplementary material for: Skin‐Inspired Thermoreceptors‐Based Electronic Skin for Biomimicking Thermal Pain Reflexes
Source: Adv Sci (Weinh). 2022 Jul 25;9(27):2201525. doi: 10.1002/advs.202201525 (PMC9507360; doi:10.1002/advs.202201525)
Supplement: Supplementary file 1 — Supporting Information [file ADVS-9-2201525-s003.pdf]

Supporting information

Skin-Inspired Thermoreceptors based Electronic Skin for Biomimicking Thermal Pain Reflexes

João Neto, Radu Chirila, Abhishek Singh Dahiya, Adamos Christou, Dhayalan Shakthivel and Ravinder Dahiya*

Bendable Electronics and Sensing Technologies (BEST) Group, University of Glasgow, Glasgow G12 8QQ, U.K

*Corresponding author: Prof. Ravinder Dahiya

Tel.: +44 (0)141 330 5653

E-mail: Ravinder.Dahiya@glasgow.ac.uk

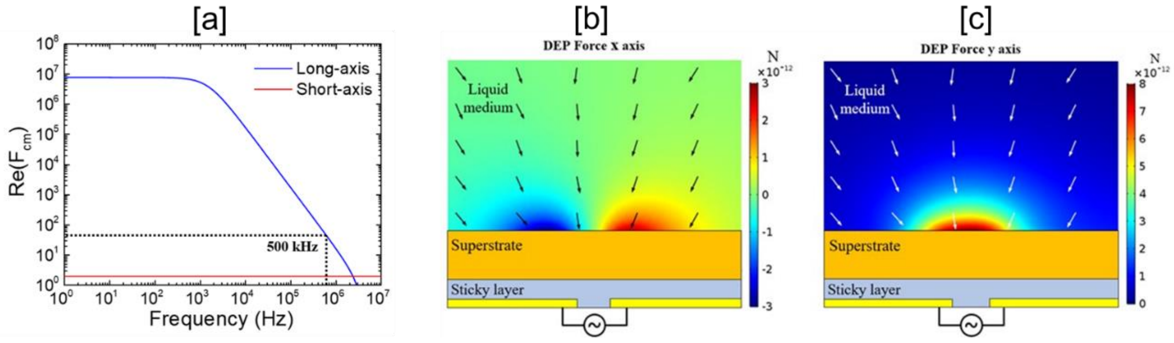

Figure S1. (a) Real part of Clausius-Mossotti plot for both short and long axis for V_2O_5 NW dispersed in DI water. (b-c) Simulated DEP force through COMSOL Multiphysics of the DEP assembly. The applied AC signal consists in 180 V and 500 kHz, both DEP force for the x and y axis field gradient are presented.

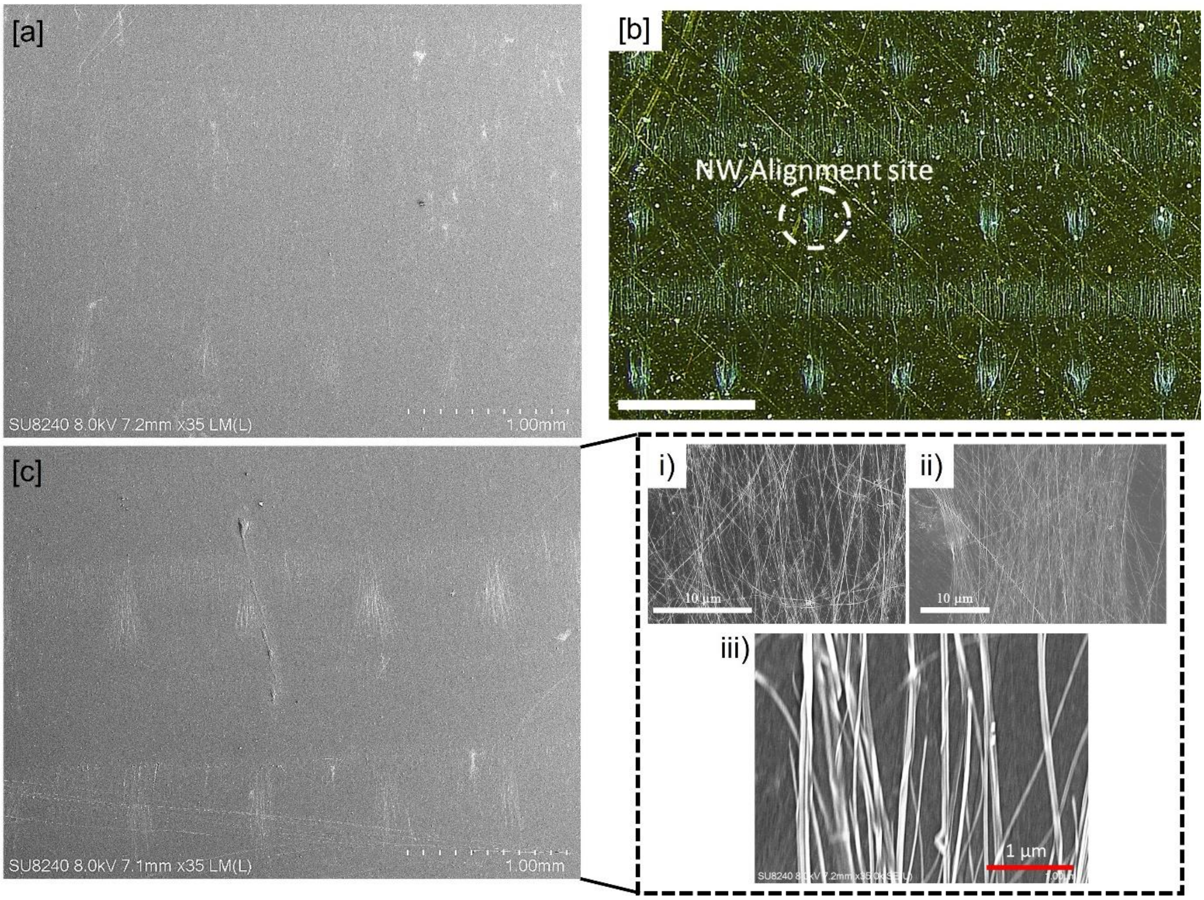

Figure S2. Optical and SEM images of the aligned V_2O_5 NWs using DEP. (a) SEM image corresponds to the DEP process carried out at 100 V_{PP} and 500 kHz. (b) Low magnification optical image corresponds to the DEP process carried out at 180 V_{PP} and 500 kHz. The scale bar in the optical image is 1.2 mm. (c) Low magnification SEM image taken from the optical image shown in figure panel b. (C (i-iii)) High magnification SEM images for SEM image shown in figure panel c.

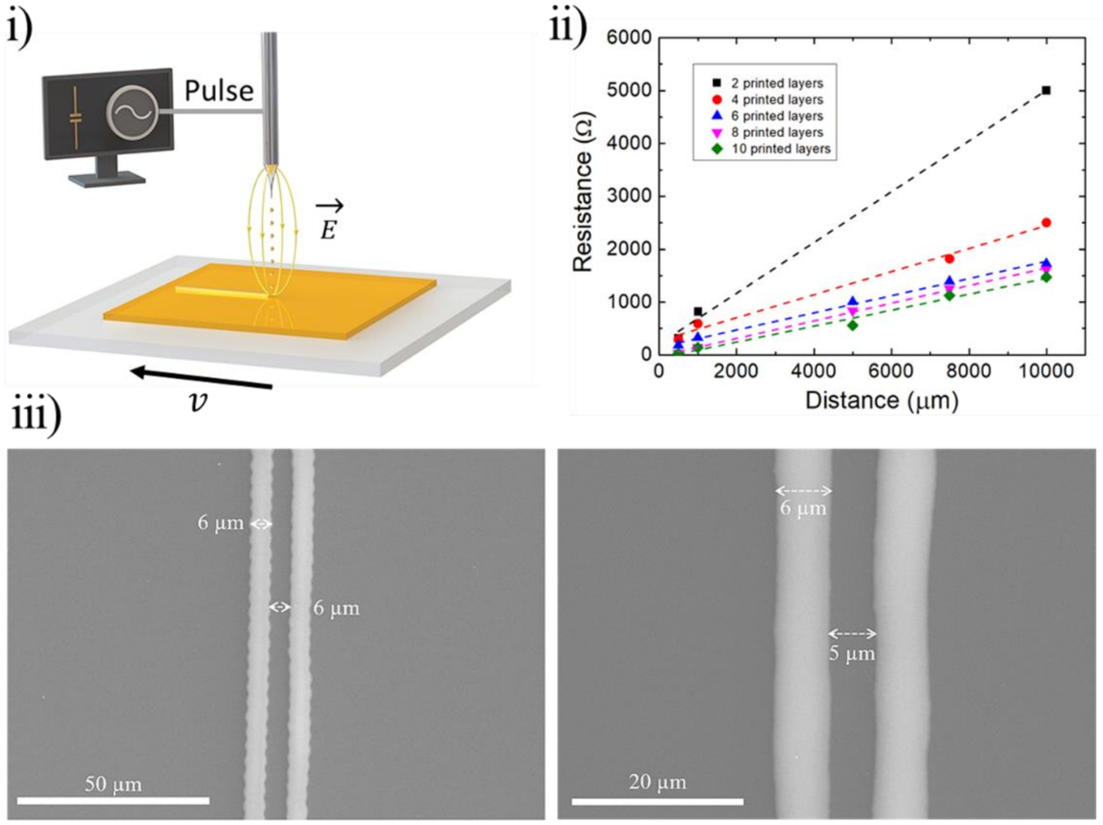

Figure S3. EHD printing optimization. (i) Schematic of the printing process, (ii) resistance vs. length graph for the printed Au layers, and (iii) SEM images showing the resolution of the Au printed lines.

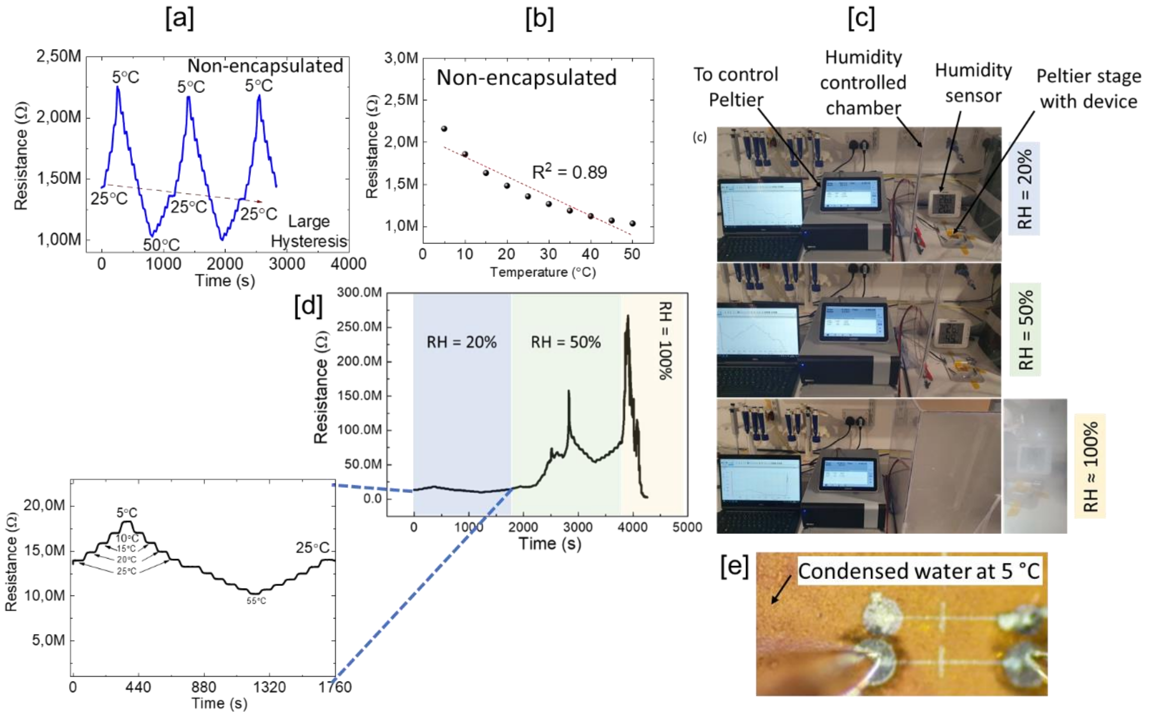

Figure S4. (a) Change in resistance with time graph for multiple heating-cooling cycles between 5-50 °C temperature range for non-encapsulated temperature sensors. The steps are 5 °C. The device showed non-linear change in resistance with step change and huge hysteresis. (b) Resistance-temperature data points showing nonlinear response from non-encapsulated sensors. (c) optical images show temperature sensors placed on Peltier stage along with humidity sensor, which displays the real time values. The humidity was controlled by dry nitrogen gas and humidifier inside the closed chamber. (d) change in resistance with time graph under various relative humidity. (e) optical image acquired during the temperature sensing measurements for non-encapsulated devices showing appearance of water at 5 °C due to condensation.

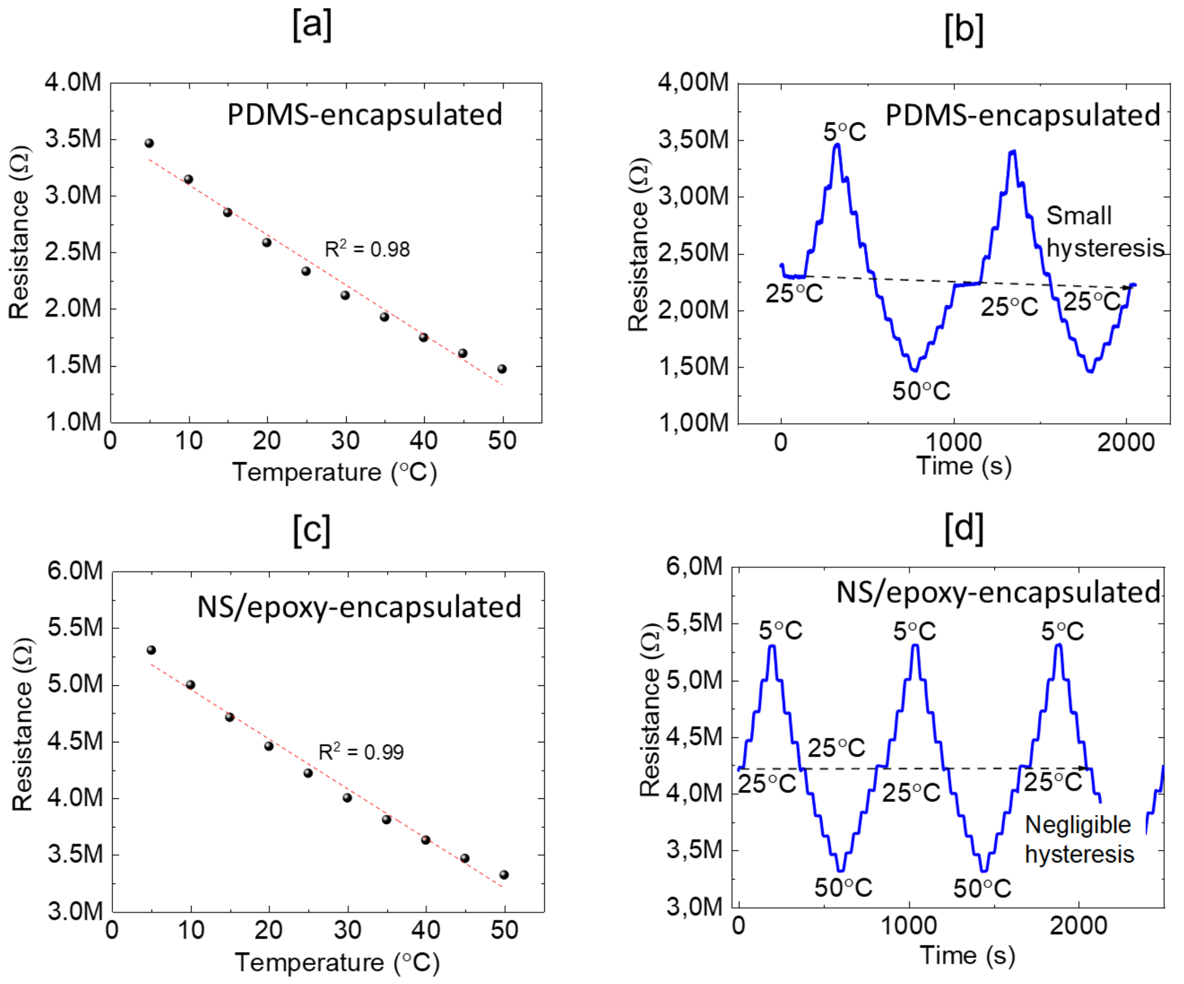

Figure S5. (a) Change in resistance with temperature for PDMS-encapsulated device. (b) change in resistance with time graph for multiple heating-cooling cycles between 5-50 °C temperature range for PDMS-encapsulated temperature sensors. The steps are 5 °C. The PDMS encapsulated sensors showed small hysteresis and non-linearity. (c) change in resistance with temperature for NS/epoxy-encapsulated device. (d) change in resistance with time graph for multiple heating-cooling cycles between 5-50 °C temperature range for NS/epoxy-encapsulated temperature sensors. NS/encapsulated sensors which showed robust sensor response with negligible hysteresis and linear response.

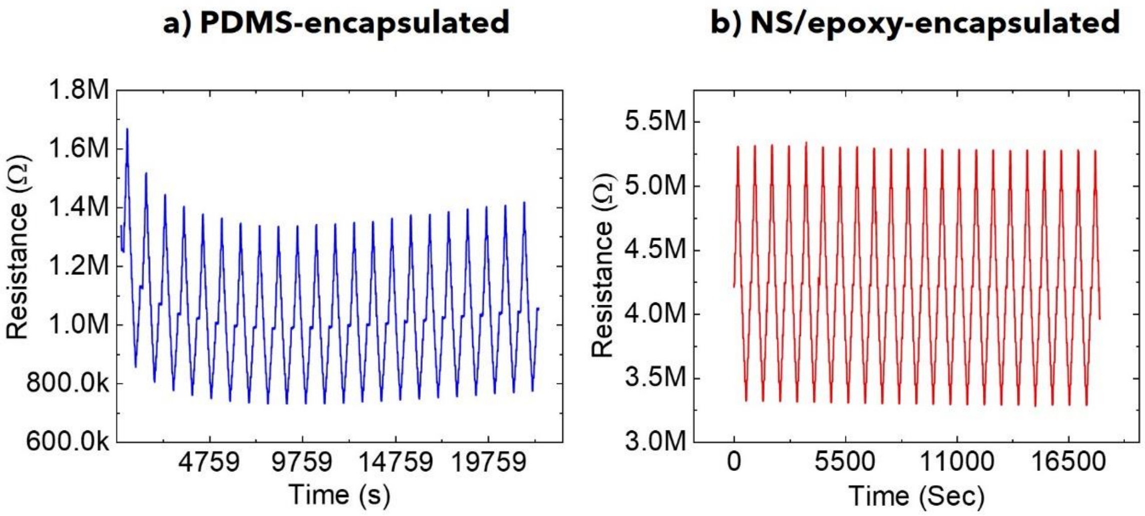

Figure S6. Continuous multiple heating-cooling cycles between 5-50 °C temperature range to monitor change in resistance for (a) PDMS-encapsulated and (b) NS/epoxy-encapsulated. The step change of temperature was 5 °C. It is evident from the data shown that NS/epoxy continue to show robust and stable change in resistance with step change in temperature.

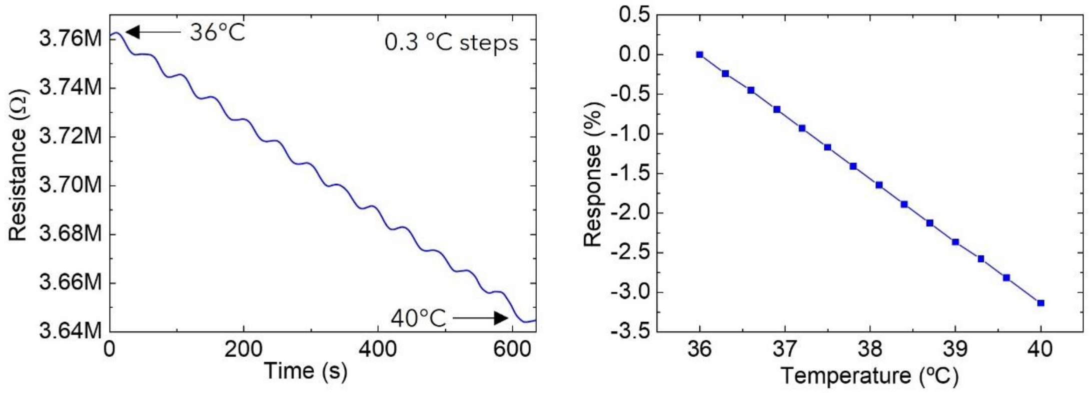

Figure S7. (a) Change in electrical resistance with temperature for NS-epoxy encapsulated device. The temperature steps are 0.3 °C. The temperature was changed from 36 to 40 °C. (b) Extracted response (%) from the data shown in Figure panel (a).

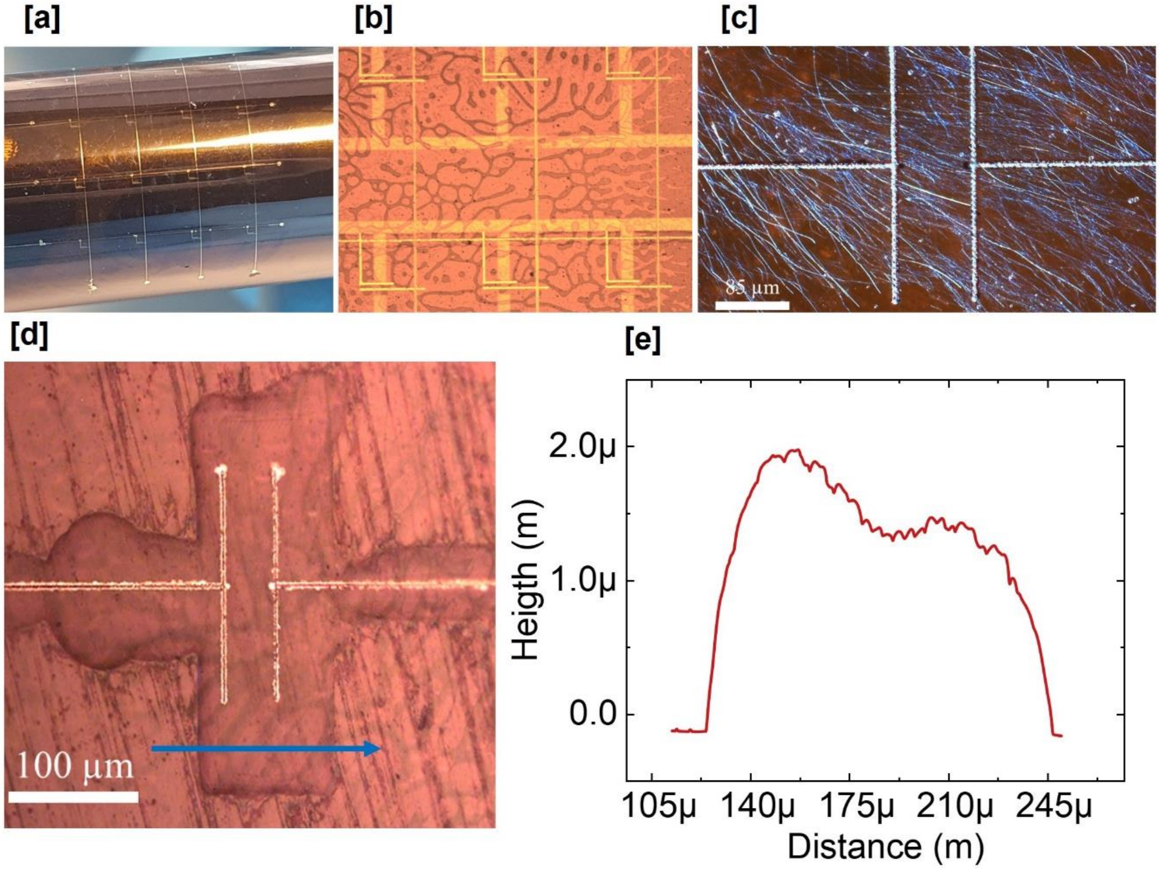

Figure S8. (a) Optical images for a fully printed 4x4 V_2O_5 NW based temperature sensing matrix placed over a glass tube showing conformal nature. (b-c) High magnification optical images of the sensors. (d) Optical image of the sensor after encapsulated with NS/epoxy and area of profilometer scan. (e) Profilometer data showing the thickness of printed NS/epoxy around 1-2 µm.

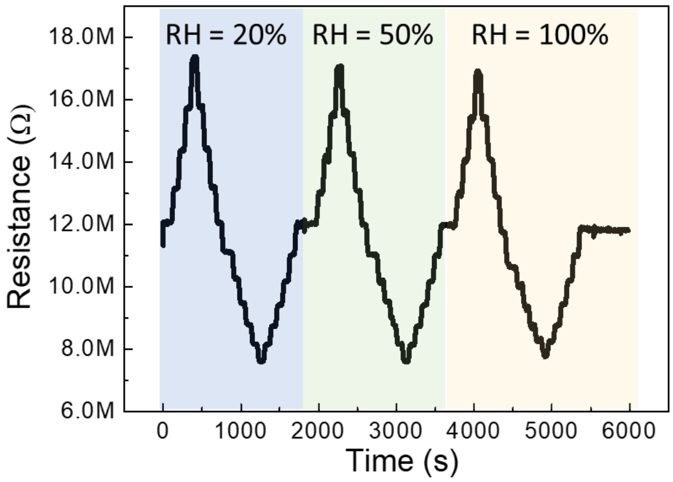

Figure S9. Influence of relative humidity (RH) on the sensing performance for devices encapsulated using NS/epoxy.

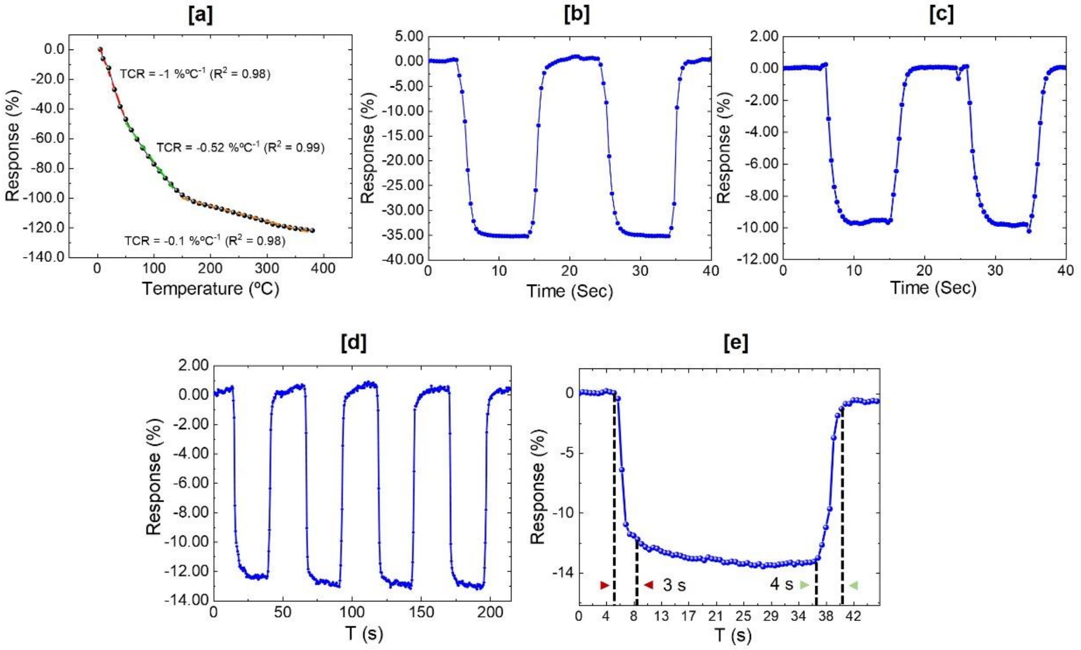

Figure S10. (a) Response (%) against temperature ranging from 5 to 375 °C for NS/epoxy-encapsulated devices. The change in resistance (b) between 5-50 °C and (c) between 28-40 °C showing no change in sensor response time for NS/epoxy-encapsulated sensors. (d-e) The change in resistance between 24-35 °C for PDMS-encapsulated sensors showing response time of 3s.

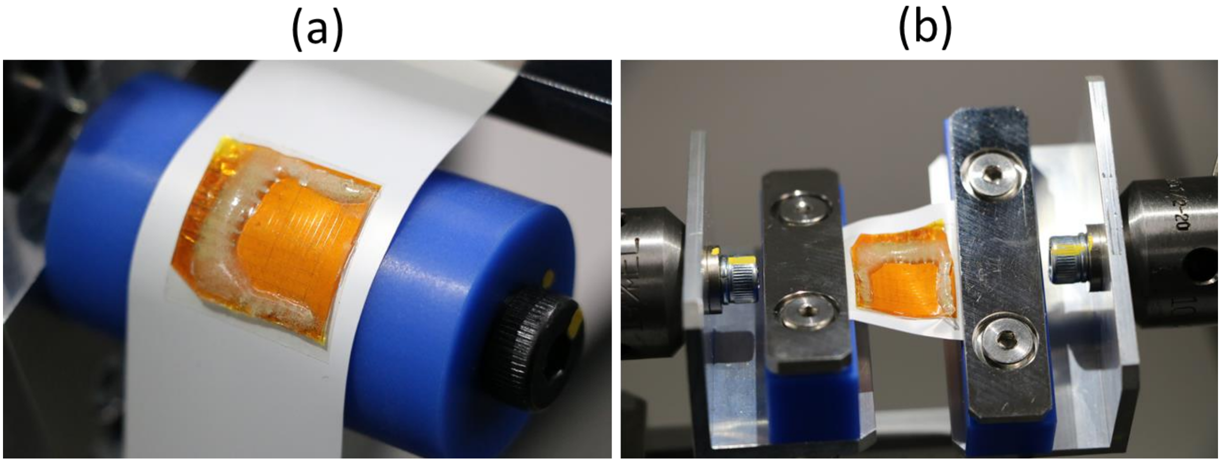

Figure S11. Full automated Yuasa endurance testing system: (a) temperature sensor matrix under bending loading (10mm), and (b) temperature sensor matrix under torsional loading (±30°).

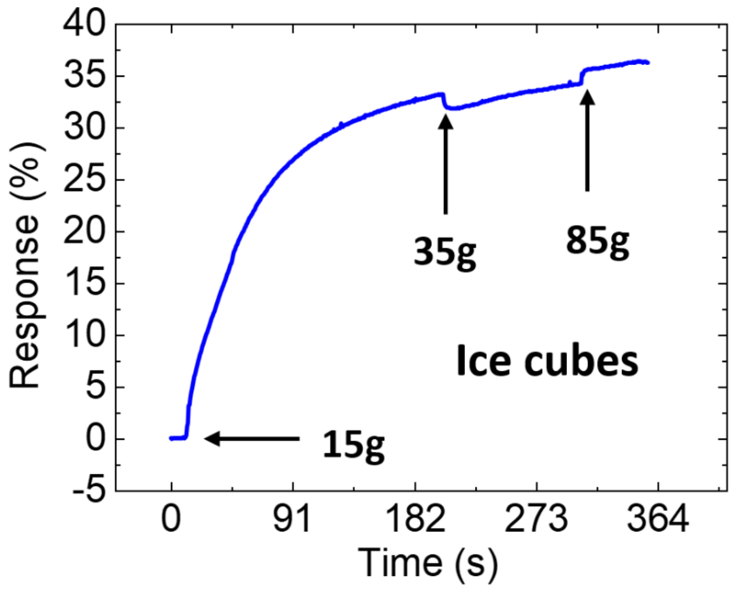

Figure S12. (i) Real-time sensor response when different weights of ice are placed on the sensor.

Supporting Note 1

Dielectrophoretic assembly of V_2O_5 NWs: The NW alignment is a two-step process. In the first, microelectrode arrays (MEA), which generate the electric field, are fabricated and the substrates assembly was prepared. Standard photolithography steps were conducted for the fabrication of MEA (as DEP electrodes). A glass slide was cleaned with acetone, IPA, and DI water under sonication bath. Positive resist was spin coated, followed by soft-bake and UV exposure and development of the electrode pattern. Then the ozone treatment was performed, and this was followed by the evaporation of 10 nm of nichrome and 100 nm of gold. In the second step, NW solution was prepared, and DEP process was performed. The V_2O_5 NWs (0.05 g) were dispersed in 10 mL of DI water to a concentration of 0.5 wt/%. The dispersion is stirred at 350 rpm for 30 minutes to detangle the clustered NWs and sonicated for 10 seconds to promote uniformity of the dispersion and fracture longer NWs into smaller lengths (<100 μm). The NW alignment assembly consists of drop casting the prepared dispersion over the substrate while applying an AC signal using a signal generator WaveStation 3082 from

Teledyne LeCroy and an amplifier A400DI from FLC electronics. The optimization details for DEP process are as follows:

The DEP force (F_{DEP}) exerted on a high aspect-ratio oblate spheroid (one-dimensional nanomaterials) can be approximated by the analytical expression[1] (eq.1):

$$F_{DEP} = \frac{2\pi(ab^2)}{3} \varepsilon_m \mathrm{Re}[K(\omega)] \nabla E^2 \qquad (1)$$

where ∇E is the gradient of the electric field, ε_m is permittivity of the medium, a is the long and b is the short radius of the ellipsoidal particle, respectively and Re [K (ω)] is given by the real part of the complex Clausius-Mossotti factor K(ω). The Clausius-Mossotti factor is divided into 2 components defined by the short and long axis, where the short axis corresponds to the radius which is responsible by the translation of the NW and the long axis by the length of the NW, which is related to the alignment of the structure with the electric field lines.[2] Both components are calculated through the following expressions[2] (eq. 2-4) which were integrated into MATLAB (See Figure S1a, Supporting Information). The vanadium pentoxide dielectric properties were defined as $\varepsilon_r = 0.05$[3] and σ = 50 S/m,[4] with length of 100 µm and 40 nm radius, the medium solvent is DI water where the dielectric properties are defined as $\varepsilon_r = 78$ and σ = 6.66E-6 S/m.

$$F_{CM-Long} = \frac{\varepsilon_P^* - \varepsilon_m^*}{\varepsilon_m^*} \quad (2) \quad \wedge \quad F_{CM-Short} = 2\,\frac{\varepsilon_P^* - \varepsilon_m^*}{\varepsilon_P^* + 2\varepsilon_m^*} \quad (3)$$

$$\varepsilon^* = \varepsilon_p - j\frac{\sigma}{\omega} \qquad (4)$$

The DEP force was simulated by integrating the DEP equation (eq.1) into COMSOL, the assembly is represented in the Supporting Information Figure S1b where gold electrodes are buried under a 5 µm PET film covered by the PI with 50 µm thickness. The electrode gap is

set at 75 µm and 180 V at 500 kHz are applied. The liquid medium is H_2O following the same dielectric constants referred above.

Supporting Note 2

Electrohydrodynamic (EHD) printing: EHD printing is employed to fabricate miniaturized and embedded temperature sensors owing to its excellent and appealing attributes that include: (i) the drop-on-demand feature, which opens the possibility for a complete additive manufacturing, thus no material wastage, (ii) possibility to print 3D structures at nanoscale, (iii) substrate independent patterning unlike photolithography and e-beam lithography, (iv) computer-controlled system, which provides the flexibility in terms of designing different structures, (v) compatibility with large area manufacturing, and (vi) the potential for low-cost operation.[5] Au contact electrodes were printed over the aligned NWs. The printed ink consists of commercial gold ink (CAu-2000 from ULVAC inc.) and NS/epoxy ink from UT Dots inc. The details for printing optimization are as follows:

Figure S3i (Supporting Information) schematically shows the EHD printing set-up. To optimize the printing, the substrate surface energy and roughness play an important role on the wettability and volume of the droplet, as well as the distance from the nozzle to the substrate. Normally in EHD systems the nozzle is located at less than 40 µm above the substrate which allows the formation of localized electric field strong enough to eject the droplets with the applied pulses leading to a good accuracy, however, such small distance may be affected by the surface roughness of the polymeric substrate, and this may lead to non-uniform printing. We also need to consider the influence of the substrate/material over which the nozzle is passing while printing. It is known that non-conductive substrates, like PI in the present case, are subject to high charge accumulation which will generate disturbances leading to spraying of the jet, thus, non-uniform printing. Difference in the electrical properties of the

semiconducting materials (V_2O_5 NW in the present case) that are assembled on the substrate could be another influencing factor. When the nozzle crosses over the NWs a slight change in the generated electric field might occur, which may lead to poor control on the droplet volume, leading to ink spreading or even shortening of electrodes which is highly likely when such small features are printed (<5 µm) over the NWs. To reduce such disturbances, a highly stable printing is required. In here, thermal and plasma treatments of the substrate were explored to remove the organic residues and humidity to allow stable printing or the passivation of the flexible substrate with inorganic oxide layers. Thermal treatment was applied to the substrate at 100ºC for 10 minutes before the printing. The printing was performed using the following optimized parameters: 75% sign wave, 300V amplitude, 0 DC bias, 80 Hz frequency and the stage moving at 0.5 mm/s. To increase the conductivity of the printed lines the same pattern was repeated 5 times. The curing step is performed at 250ºC for 2 hours.

The resistance of printed Au lines with the number of printed layers is extracted following the electrical characterization. By increasing the number of Au layers, the cross-sectional area of metal and material volume was increased to reduce the resistance. Figure S3ii (supporting information) shows a linear behavior of the metal lines according to the distance where from 6 to 10 printed layers the conductivity starts to saturate. However, increasing the printed layers will also influence the resolution and definition of the printed feature, the previously printed ink may retain charges which will affect the droplet volume of the layers printed on top, leading once more to undesired patterning/spraying effects. A careful set of parameters must be chosen to guarantee well-defined metal lines while maintaining a good level of conductivity which does not influence the performance of the device. The optimized EHD printing parameters allow a channel length of about 5 µm (Figure S3iii).

Supporting Note 3

Sensor response: The sensitivity of semiconductor-based temperature sensors is defined by the temperature coefficient of resistance (TCR), which expresses the relative change of a temperature sensors' resistance R when the temperature T changed by a unit, as shown in formula below:[6]

$$TCR = \frac{1}{R}\frac{dR}{dT} = \frac{(R-R_0)}{(T-T_0)R} \qquad (5)$$

where dR denotes the change in R, with a temperature change of dT. R_0 is the nominal resistance of temperature sensor at room temperature T_0. R is the resistance at temperature T.

The sensor response (%) for all three variants was measured under identical conditions and is given by:

$$Response = \left(\frac{\Delta R}{R_0}\right) \times 100 \qquad (6)$$

Where ΔR is the change in the resistance, and R_0 is the base resistance.

Supporting Note 4

Charge transport: The charge transport in V_2O_5 is defined by electron hopping between V^{5+} and V^{4+} impurity centers. This plot is used to analyses the data in the frame of the general formula for electrical conductivity of transition-metal oxides proposed by Mott, where the conductivity is given by:[7]

$$\sigma = \left(\frac{\vartheta_0 e^2 C(1-C)}{kTR}\right) \exp(-2\alpha R) \exp(-\frac{W}{kT}) \qquad (7)$$

where ϑ_0 is a phonon frequency, C the concentration ratio $V^{4+}/(V^{4+} + V^{5+})$, R the average hopping distance, W the activation energy, T the temperature, and α the rate of wave function decay.

Supporting Note 5

Neural network to train robots to feel, learn and react to external heat stimuli: Perception of the thermal environment begins with the activation of peripheral hot and cold thermal receptors. This step is performed by sensing the change in printed sensor resistance using a 16-bit analog-to-digital converter (ADC). The output voltage of a potential divider was read, and the resulting data stream was fed into a pre-processing pipeline. The data stream was filtered

using an α-β filter (implemented on the microcontroller) and output is fed into the neural classifier. The neural network is a simple 2-perceptron classifier, able to distinguish between three temperature ranges: (i) Cold – less than the normal skin-temperature, (ii) skin temperature, and (iii) hot – higher than skin-temperature. Training of this neural network was performed using backpropagation, adjusting both the weights of the neurons and the threshold of their activation functions. The details are as following:

Data readout

Since the temperature sensor is resistive, the raw data was read using a 16-bit ADC. The output voltage of a potential divider was read, and the resulting data stream was fed into a pre-processing pipeline.

Pre-processing

To minimise the noise of the system and get the most accurate temperature readout, the data stream was filtered using an α-β filter (implemented on the microcontroller) and the output was averaged over 200 samples. The first 200 datapoints are taken as a reference for the room temperature and the reference resistance is thus established for the circuit. The following datapoints, pre-processed in the same way, are used to compare the subsequent change in temperature and output the final temperature value, which is then fed into a neural classifier.

Neural network architecture

The neural network is a simple 2-perceptron classifier, able to distinguish between 3 temperature ranges:

- Cold – less than normal room temperature range,
- Room temperature,
- Hot – higher than room temperature.

Since a perceptron is a binary classifier used in supervised learning, the network had to contain 2 perceptual units, driven by the same input. Each neuron's behaviour was defined by a Heaviside activation function, resulting in the following transfer relationship:

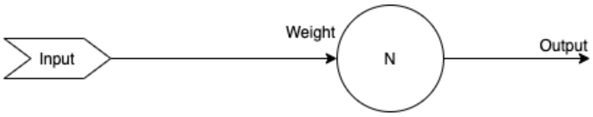

$$\hat{y} = \begin{cases} 0, \sum input * weight < threshold \\ 1, \qquad otherwise \end{cases}$$

Training of this network was done using backpropagation, adjusting both the weights of the neurons and the threshold of their activation functions. The relationships describing the training steps are the following:

$$new\_w = current\_w + learning\_rate * (correct\_output - current\_output) * input$$

$$new_{threshold} = current\_threshold - learning\_rate * (correct\_output - current\_output)$$

where the learning rate is a constant which defines how much can the weights and thresholds vary after each training step.

The output is controlled by a classification function f(N1,N2):

$$f(N1, N2) = \begin{cases} 0, & if\ N1 = 0 \\ N1 + N2\,, & otherwise \end{cases}$$

[network figure] [important to note that this all was done to get a biomimetic behaviour]

Classification and training

Since there are 2 neural units and 3 classes, the classical classification approach using argmax (output layer) is not possible. In this case, the output can be interpreted as a binary relationship between temperatures and thresholds. The first perceptron outputs 1 only if the input is larger than the upper limit of the first class. Similarly, the second perceptron outputs 1 if the temperature is higher than the lower limit of the third class. This results in an implicit, perceptually learned definition of the second class.

Training data was lightweight, and each perceptron was trained via backpropagation separately. The dataset for each perceptron (N1, and N2) is shown below:

Input (temperature)	Perceptron output
10	0
22	0
24	1
50	1

Neuron 1 training dataset

Input (temperature)	Perceptron output
27	0
35	0
38	1
55	1

Neuron 2 training dataset

The possible outputs in this case are the following:

N1	N2	F(n1,n2)
0	0	0
0	1	N/A
1	0	1
1	1	2

Training of the neural network was performed over 300 epochs, with a learning rate of 1, to ensure the stability of the learned thresholds. Since the neural units are perceptron, and the classes are well defined, overfitting of the network during training ensures there is no ambiguity in the case of the second class (room temperature).

Table S1. Performance comparison of the state-of-the-art printed temperature sensors

**Sensing material**	**Functional material assembly method**	**Sensitivity (% °C^{-1})**	**Sensing Range (°C)**	**Response/ Recovery Time (s)**	**Neuromorphic data processing demonstrated**	**Area (mm^2)**	**Ref.**
PEDOT:PSS /CNT	Screen	0.63	25 – 53	–	No	12	[8]
Ag NP	Inkjet	0.108	0 – 100	–	No	49	[9]
Ag NP	Inkjet	0.22	20 – 60	–	No	644	[10]
Graphene/ PEDOT:PSS	Inkjet	0.06	35 – 45	20 / 18	No	1.34	[11]
CNT	Gravure	- 0.4	- 40 – 100	0.3 / 4	No	1.94	[12]
GO	Inkjet	1.48	25 – 85	0.5 / 10	No	64	[13]
NiO	Inkjet	-	50 – 200	1 / 360	No	0.25	[14]
V_2O_5 NW	DEP	- 1.1	5 – 50	1 / 2	Yes	0.0007	Present work

References

[1] H. Morgan, N. G. Green, *Journal of Electrostatics* **1997**, 42, 279.
[2] Y. Liu, J.-H. Chung, W. K. Liu, R. S. Ruoff, *J. Phys. Chem. B* **2006**, 110, 14098.
[3] R. Sengodan, B. C. Shekar, S. Sathish, *Physics Procedia* **2013**, 49, 158.
[4] J. Muster, G. T. Kim, V. Krstić, J. G. Park, Y. W. Park, S. Roth, M. Burghard, *Adv. Mater.* **2000**, 12, 420.
[5] a) Q. Lei, J. He, D. Li, *Nanoscale* **2019**, 11, 15195; b) P. Galliker, J. Schneider, H. Eghlidi, S. Kress, V. Sandoghdar, D. Poulikakos, *Nat Commun* **2012**, 3, 890; c) J.-U. Park, M. Hardy, S. J.

Kang, K. Barton, K. Adair, D. k. Mukhopadhyay, C. Y. Lee, M. S. Strano, A. G. Alleyne, J. G. Georgiadis, P. M. Ferreira, J. A. Rogers, *Nature Mater* **2007**, 6, 782.
[6] X.-F. Zhao, S.-Q. Yang, X.-H. Wen, Q.-W. Huang, P.-F. Qiu, T.-R. Wei, H. Zhang, J.-C. Wang, D. W. Zhang, X. Shi, H.-L. Lu, *Advanced Materials* **2022**, 34, 2107479.
[7] N. F. Mott, *Journal of Non-Crystalline Solids* **1968**, 1, 1.
[8] S. Harada, W. Honda, T. Arie, S. Akita, K. Takei, *ACS Nano* **2014**, 8, 3921.
[9] S. Ali, A. Hassan, J. Bae, C. H. Lee, J. Kim, *Langmuir* **2016**, 32, 11432.
[10] M. D. Dankoco, G. Y. Tesfay, E. Benevent, M. Bendahan, *Materials Science and Engineering: B* **2016**, 205, 1.
[11] T. Vuorinen, J. Niittynen, T. Kankkunen, T. M. Kraft, M. Mäntysalo, *Scientific Reports* **2016**, 6, 35289.
[12] V. S. Turkani, D. Maddipatla, B. B. Narakathu, B. J. Bazuin, M. Z. Atashbar, *Sensors and Actuators A: Physical* **2018**, 279, 1.
[13] D. Kong, L. T. Le, Y. Li, J. L. Zunino, W. Lee, *Langmuir* **2012**, 28, 13467.
[14] C.-C. Huang, Z.-K. Kao, Y.-C. Liao, *ACS Applied Materials & Interfaces* **2013**, 5, 12954.
